# Impulsivity, Emotional Intelligence, and Alcohol Consumption in Young People: A Mediation Analysis

**DOI:** 10.3390/foods9010071

**Published:** 2020-01-08

**Authors:** Ana Merchán-Clavellino, María Pilar Salguero-Alcañiz, Rocío Guil, Jose Ramón Alameda-Bailén

**Affiliations:** 1 Department of Psychology, Faculty of Education Sciences, University of Cádiz, 11519 Puerto Real, Spain; rocio.guil@uca.es; 2 INDESS (Research Universitary Institute for Sustainable Social Development), University of Cádiz, 11406 Jerez de la Frontera, Spain; 3 Basic Psychology Area, Department of Clinical and Experimental Psychology, University of Huelva, 21007 Huelva, Spain; pilar.salguero@dpsi.uhu.es (M.P.S.-A.); alameda@uhu.es (J.R.A.-B.)

**Keywords:** alcohol, impulsivity, emotional intelligence, sensation seeking

## Abstract

Alcohol consumption in young people is a public health problem. Due to the harmful consequences and the large population using alcoholic substances, it would be important to determine the biological, psychological, and social factors associated with alcohol use and abuse. The main object of this study is to explore which components of impulsivity, according to the main theoretical models, have predictive power regarding alcohol consumption in young people. A secondary objective is to determine if emotional intelligence has a mediating role between the components of impulsivity and alcohol consumption, and thus specifically contribute to the knowledge about the mediation processes between those variables that are involved in the initiation and maintenance of alcohol consumption. For this purpose, 384 participants were recruited (83.1% females, *n* = 319), with mean age of 20.46 years (*SD* = 1.90; range 18–25). All participants were alcohol consumers at the beginning of the study. Data collection was conducted via online survey; impulsivity was evaluated by several questionnaires (Sensation Seeking Scale Form V, Barratt Impulsivity Scales 11, and Sensitivity to Punishment and Sensitivity to Reward), and emotional intelligence was evaluated by the Trait Meta-Mood Scale. The results reveal that the dimension of disinhibition (a dimension of the sensation search scale) significantly has the highest predictive value on alcohol consumption. Moreover, our data show that the total effect and direct effect of disinhibition on frequency of alcohol consumption were both significant. The mediating role of emotional intelligence in this process was also significant. These findings show which variables should be considered to prevent alcohol consumption in young people.

## 1. Introduction

Alcohol consumption in young people is a public health problem, as recognized by the World Health Organization (WHO) [1]. Around the world, 26.5% of young people between 15–19 years old consume alcohol, representing 155 million adolescents, with the highest consumption rates currently in Europe, specifically 43.8% [1,2].

Therefore, the future impact on the health of young people is enormous, in such a way that it would imply the increase of possible diseases, such as certain types of cancer, cardiovascular dysfunction, and liver problems [3] associated with premature deaths [1]. Due to the harmful consequences and the large population that uses these substances, different disciplines, including psychology, have joined forces to determine the biological, psychological, and social factors that are associated with alcohol use and abuse.

From a psychological point of view, evidence indicates that personality variables are clearly associated with alcohol consumption. Indeed, numerous works have related different patterns of alcohol consumption with different personality traits [4,5,6,7]. Considering the great variety of personality traits, the purpose of the present study is to explore which personality traits, specifically impulsive traits, have a greater predictive power on alcohol consumption in young people.

Additionally, alcohol consumption has been linked to impulsivity. Previous research has consistently demonstrated the relationship between impulsivity and alcohol consumption in young people, showing that greater impulsivity is associated with higher alcohol consumption [8,9,10,11,12]. However, there is no consensus on how impulsivity could be defined and measured [11,13]. Different models and theories have proposed different conceptualizations [14,15,16,17], but the concept that impulsivity is a multidimensional construct is nowadays the most accepted [18]. Thus, it seems necessary to investigate which of the characteristics or dimensions of impulsivity have a greater impact on alcohol consumption.

Different studies are based on the main theoretical models of impulsivity and their relationship with alcohol and other substance abuse. For example, based on the personality model of the five factors, Shin et al. (2012) [19] investigated the potential of the four sub-traits of impulsivity (urgency, lack of premeditation, lack of perseverance, and search for sensations) to influence different patterns of alcohol consumption (frequency, alcohol-related problems, excessive consumption, and alcohol use disorders). These authors concluded that the search for sensations and urgency are consistently related to all alcohol consumption patterns. Particularly, they found that people with high urgency and search for sensations have the highest levels of alcohol consumption, as well as problems related to alcohol consumption. They also found that the lack of premeditation is associated with alcohol consumption, but not with alcohol-related problems. Therefore, different dimensions of impulsivity could have different effects on alcohol consumption patterns. Likewise, Aluja et al. (2019) [20], in a sample of men, studied the effects of personality traits in drinkers, but in this case based on the personality models of Eysenck, Gray, and Zuckerman. They showed that the impulsive-inhibited personality factor is related to alcohol consumption, as well as to alcohol-related problems. Therefore, greater impulsivity and disinhibition could be associated with greater alcohol consumption.

Overall, it seems that impulsive personality traits could be optimal predictors of different alcohol consumption patterns. We hypothesize that the dimension of disinhibition could be considered the impulsiveness trait with the greatest predictive value over alcohol use in young people, according to Aluja et al. [20] who found that the impulse-disinhibition factor was strongly related to alcohol.

However, alcohol use is a multifactorial factor and, in addition, should be considered the indissoluble union between cognition and emotion [21]. Therefore, we hypothesize that emotional processes may act as mediators in the relation of the different aspects of impulsivity and alcohol consumption in young people.

Specifically, the concept that could reflect this union is emotional intelligence (EI). This is also supported by previous studies that have linked this construct with the use of several substances. Currently, the most accepted definition considers the EI as “the ability to perceive, evaluate and express emotions accurately, the ability to access and/or generate feelings when they facilitate thinking; the ability to understand emotions and emotional knowledge, and the ability to regulate emotions by promoting emotional and intellectual growth” [22] (p. 5). With respect to the measurement process, one of the most used questionnaires is the Trait Meta-Mood Scale (TMMS-24), which measures the level of emotional self-efficacy [23], a thorough ability to identify one’s emotions and those of others and know how to express them (emotional attention), to understand emotions (emotional clarity), and to handle emotions (emotional repair).

Considering this background, the relationship between EI and alcohol consumption has also been analyzed in this study. EI has been shown to correlate negatively with alcohol and tobacco consumption in adolescents. Therefore, young people showing adequate EI can interpret the emotions of others and detect unwanted group pressure, revealing that EI is able to generate greater resistance to alcohol and tobacco consumption [24,25]. EI is also related to the use of other substances, such as cannabis [26], which suggests that a low EI is not only predictive of alcohol consumption but also of the abuse of other drugs [27,28,29,30].

In addition, some studies confirmed the relationship between EI and impulsivity. According to some results, a low EI, that is, a poor reasoning capacity on one’s own or another’s mood, could provoke more impulsive responses to situations of threat or frustration. Therefore, improving emotional clarity (understanding of emotions) and emotional repair or regulation (ability to handle emotions) components could improve impulsive behavior [31].

To our knowledge, there are no data on the relationship between EI, impulsivity, and alcohol consumption, although the influence of these variables has certainly been described regarding cannabis use. Particularly, it has been showed that impulsive young people tend to abuse cannabis more often, and EI appears to be related to consumption, since young people focused on their own emotions and lacking adequate mechanisms to control them are prone to the excessive consumption of cannabis as a coping mechanism [32].

In summary, it can be argued that both impulsivity and EI are related to alcohol consumption. Therefore, EI, considered as the ability to process emotional information for adaptive purposes, could be a mediating variable regarding the effects of impulsivity on alcohol consumption.

**Figure 1:**
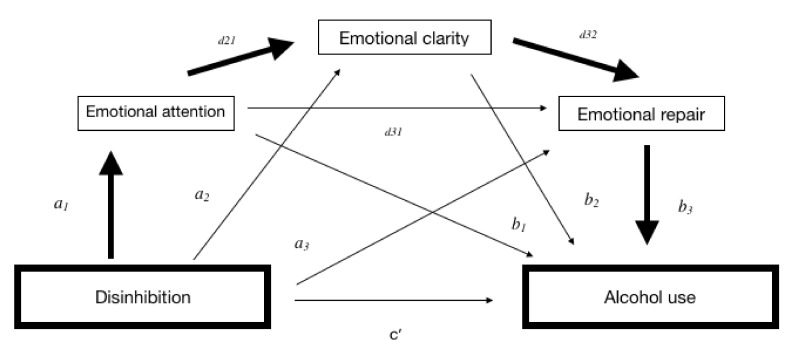
Representation of the indirect effects for serial mediation. **Notes:** direct effect (*c*’); total indirect effect (*a*) represents the association between the predictors DIS and three mediators (*a*_1_, *a*_2_, and *a*_3_); total indirect effect (*b*) refers to the role of the three mediators in the use of alcohol (*b*_1_, *b*_2_, and *b*_3_); total indirect effect (*d*) refers to the relationship of the three mediators with each other (*d*_21_, *d*_32_, and *d*_31_).

Thus, the objectives of this study are: (i) to explore what components of impulsivity, according to the main theoretical models (proposed by authors such as Zuckerman, Barrat and Gray) [14,15,16,17] have predictive value over alcohol consumption in young people, and (ii) to analyze if EI (attention, clarity and emotional repair) has a mediating effect between the components of impulsivity and alcohol consumption. Our hypothesis is that greater impulsiveness will be associated with lower levels of EI, that is, an inadequate attention level, which implies a poor compression of emotions, and therefore an inadequate capacity to regulate them, which, in turn, implies greater alcohol consumption, both in frequency and quantity (see Figure 1 for a representation of these interactions).

In summary, this study could have an impact on global public policies, since they could reveal some psychological determinants of abuse behavior in young people. More interesting, the results could help to clarify the processes that arise between variables that initiate and maintain the use of drugs, providing a view to designing prevention programs that can be developed through personalized profiles.

## 2. Materials and Methods

### 2.1. Participants

The sample consists of 384 Spanish university students (83.1% females, *n* = 319) with a mean age of 20.46 years (*SD* = 1.90; minimum = 18; maximum = 25). All participants were consumers of alcohol, and reported an average consumption in the last year of 48.33 times (*SD* = 44.76; minimum = 1; maximum = 360) and an average amount of consumption of 3.43 units (*SD* = 1.64; minimum = 0.5; maximum = 10) on the day of consumption.

### 2.2. Procedure

Participation in this study was voluntary and confidential. The study was conducted in compliance with the Declaration of Helsinki of 1975, and all participants signed the informed consent. All students completed the online self-report questionnaires and their participation was rewarded with course credits.

### 2.3. Instruments

The following questionnaires were used for the evaluation of impulsivity:

Sensation Seeking Scale (SSS-V) [14]. A Spanish adapted version consisting of 42 items of forced-choice was used. This version assesses four aspects of sensation seeking: Thrill and Adventure Seeking (TAS), Experience Seeking (ES), Disinhibition (DIS) and Boredom Susceptibility (BS). In our sample, a total alpha of 0.8 was obtained, and for each subscale the following alpha values were observed: TAS = 0.8; ES = 0.5; DIS = 0.7; BS = 0.5. 

Barratt Impulsivity Scales 11 (BIS-11) [33]. The Spanish version of Oquendo, Baca-García, Graver, Morales, Montalbán, and Mann (2001) [34] scale was used. This version consists of 30 Likert 1-4 scale items and covers the three dimensions of impulsivity proposed by Barratt: Cognitive (CI), Motor (MI), and Non-planning (NPI). The reliability of the scale is adequate, showing a total alpha of 0.6, 0.4 for the subscale CI, 0.6 for the MI, and 0.6 for the NPI subscales.

The Sensitivity to Punishment and Sensitivity to Reward Questionnaire (SPSRQ) [35]. This is a Spanish version to evaluate the behavioral inhibition system (BIS) and the behavioral approach system (BAS) [36]. This version consists of 48 dichotomous items (Yes, No) and is divided into two scales: Sensitivity to Punishment (SP), which consists of 24 items considered measures of BIS, and Sensitivity to Reward (SR) as a measure of BAS. The reliability of the scale is adequate, with the SP scale showing an alpha of 0.83 and the SR scale showing an alpha of 0.76 [37]. In our sample of young people, Cronbach’s alphas are similar, since the SP scale shows an alpha of 0.8, and the SR scale has an alpha of 0.7.

On the other hand, the Spanish version [38] of the Trait Meta-Mood Scale (TMMS-24) [23] was used for the evaluation of Emotional Intelligence. This questionnaire assesses the perception or beliefs about one’s emotional abilities. It contains 24 items, rated on a 1–5 Likert scale. TMMS-24 is divided into three dimensions, each consisting of 8 elements: emotional attention (ability to identify one’s emotions and those of others and know how to express them), emotional clarity (understanding of emotions) and emotional repair or regulation (ability to handle emotions). The reliability and validity indexes reported are adequate [38]. In our sample the alpha value was 0.8 for the total scale, 0.8 for the attention dimension, 0.9 for clarity, and 0.8 for emotional repair.

Finally, an ad hoc questionnaire was applied which included items about sex (man and woman), age, and alcohol consumption in the last year, where the frequency of alcohol consumption was recorded by the days of consumption in a year, and where the consumption amount was measured through the number of drinks in one day of consumption.

### 2.4. Statistical Analysis

All analyses were carried out using the SPSS package (version 20.0; IBM, Chicago, IL, USA). Descriptive statistics and the Student’s t-test were used to determine sex differences as described in the preliminary analyses. In addition, Pearson correlations were calculated for all study variables, including age. To verify the predictive value of personality models on over alcohol consumption (frequency and quantity), multiple regression models were performed including sex as control variable. From the predictive models, the mediation analyses were performed with the PROCESS macro [39]. We used model 6 to examine the direct and indirect effects of impulsivity on alcohol consumption (frequency). Mediation analyses were completed using EI as mediator. To verify which of the indirect effects was the most influencial, we conducted specific contrasts for indirect effects. As a criterion of statistical significance, we used the 95% confidence interval (CI) generated by the bias-corrected bootstrap method set to 10,000 reiterations.

## 3. Results

### 3.1. Preliminary Analyses

The descriptive statistics of each study variable are presented, both for the total sample and separately for men and women (Table 1). Analyses of the differences between sex are also included.

Significant differences in the factor impulsivity were observed using the above-mentioned questionnaires. According to the SSS-V, men obtained significantly higher scores in the TAS and DIS dimensions. The results obtained in the BIS-11 scale are congruent, showing that the scores of men were significantly higher than those of females in the CI and NPI dimensions. Based on the Gray model (SPSRQ), male scores were significantly higher than those of women in SR, but not in SP. With respect to emotional intelligence, there are no differences between men and females in any of the three dimensions assessed by the TMMS-24.

Table 2 shows the Pearson correlations between the main variables of the study. The results indicate a relationship between impulsivity and alcohol consumption, with different variations depending on the instrument used in the impulsivity evaluation. Specifically, the SSS-V shows positive correlations with alcohol consumption for the dimensions TAS, ES, and DIS. The results of the BIS 11 also reveal significant positive correlations between both non-planning and motor impulsiveness. Likewise, the results of the SPSRQ show a significant correlation between the sensitivity to reward and alcohol consumption.

Regarding the variable EI (Table 2), we found a significant negative correlation between alcohol consumption and emotional attention. We also observed that emotional dimensions correlate positively each other, that is, attention with clarity and clarity with emotional repair.

To explore what dimensions of impulsivity could best predict alcohol consumption, based on the significant correlations observed, multiple linear regression analyses were conducted as described above. The variable sex was included in both analyses as a control variable.

Table 3 shows the results of multiple regression analyses; model 1 has all subscales of impulsivity and emotional intelligence, model 2 has all subscales and sex, model 3 includes only the significant subscales, and model 4 includes significant subscales and sex. We conducted analyses for the frequency of alcohol consumption and for the amount of alcohol consumed. 

All the models revealed significant effects (*p* = 0.000) with interval adjusted *R*^2^ from 0.118 to 0.132 and of all impulsivity subscales, only DIS was associated with the frequency of alcohol consumption (*p* < 0.01). Regarding the amount of alcohol consumed, the analyses showed significant effect (*p* = 0.000), with adjusted *R*^2^ from 0.072 to 0.091. In congruence with the previous model, the results show that disinhibition is the only variable significantly associated with the amount of alcohol consumed (*p* < 0.01).

### 3.2. Mediation Analyses

In the present study, DIS was considered the first variable (predictor, X) and the frequency of alcohol consumption was the measured result (Y). Emotional attention (M1), emotional clarity (M2), and emotional repair (M3) were considered mediating variables. In the second model (according to the amount of alcohol consumed), the mediation of indirect effects was not considered relevant to be analyzed because no correlations between the amount of consumption and any of the dimensions of emotional intelligence were found.

As illustrated in Figure 1, total effect (*c*) refers to the relationship between disinhibition and alcohol, in terms of Frequency, without controlling for the mediators; direct effect (*c*′) refers to the relationship between DIS and Frequency, after controlling for the mediators; total indirect effect (*a*) represents the association between the predictors DIS and three mediators (*a*_1_, *a*_2_, and *a*_3_); and total indirect effect (*b*) refers to the role of the three mediators in the frequency of consumption (*b*_1_, *b*_2_, and *b*_3_). Total indirect effect (*d*) refers to the relationship of the three mediators with each other (*d*_21_, *d*_32_, and *d*_31_), and specific indirect effect (*a*_1_*b*_1_, *a*_2_*b*_2_, and/or *a*_3_*b*_3_) refers to the role of a specific mediator in the relationship between DIS and frequency.

The model that evaluates the possible mediation of EI (attention, clarity, and repair) in the relationship between DIS and frequency is shown in Table 4. In the first regression, DIS accounted for 9.71% of the unique variance in frequency (*R*^2^ = 0.0971, *F* = 41.06, *p* < 0.01). However, 12.39% of the total amount of variance was accounted for by the global model, which included DIS and the three proposed EI mediators (*R*^2^ = 0.1205, *F* = 12.98, *p* < 0.01).

**Figure 2:**
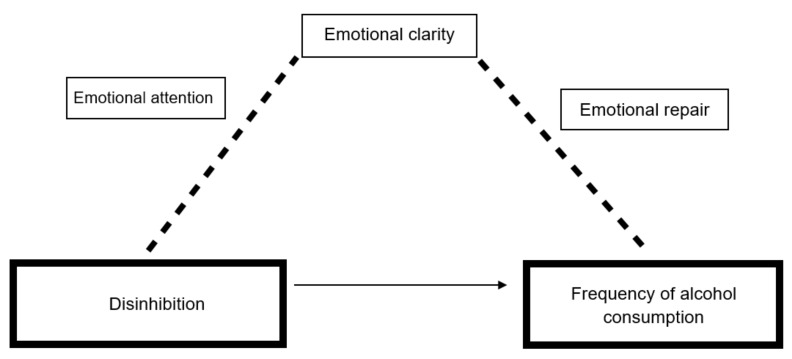
Illustration of mediation model between disinhibition and frequency of alcohol consumption.

The values provided in Table 4 show that the total effect (*c*) and the direct effect (*c*’) of DIS on Frequency were both significant. According to the regression coefficient, based on the fact that the 95% CI of the point estimate does not contain zero—which is evidence of the mediation of indirect effects, we obtained one specific indirect effect through the emotional clarity relationships (Ind5 = *a*_2_*b*_2_), in which less DIS was associated with greater emotional clarity which was, in turn, associated with higher frequency of alcohol consumption. Therefore, it can be argued that EI, through clarity, mediates the relationship between impulsivity (disinhibition) and the frequency of alcohol consumption (Figure 2).

## 4. Discussion

The first objective of this study was to determine which component of impulsivity shows a greater impact on alcohol consumption in young people. Three impulsiveness measures were used for this purpose, according to several main models based on: (a) SSS-V-Zuckerman [14], (b) BIS-11-Barrat [15,33], and (c) SPSRQ-Reinforcement Sensitivity Theory-Gray [16,36].

Our results show significant differences in the impulsivity variable between females and males, as revealed by different questionnaires (SSS-V, BIS-11, and SPSRQ), with higher scores observed in males. These results are congruent with previous studies that reveal higher impulsiveness scores (and sensation seeking) in men compared to women [40,41].

On the other hand, our results show correlations between the different dimensions of impulsivity and alcohol consumption. The results of the SSS-V scale indicate that alcohol consumption correlates with the search for sensations, emotions, adventures, search for experiences and disinhibition subscales. These results confirm the previously described association between impulsivity and alcohol consumption in adolescence [42]. Specifically, the search for sensations has been strongly associated with the frequency of consumption [20,43,44,45], although this variable is also relevant in other aspects of alcohol consumption, such as excessive alcohol consumption and alcohol-related problems [20].

Similarly, and according to the model proposed by Barrat in 1985 [15], alcohol consumption correlates with non-planning impulsivity, but not with the dimensions of cognitive and motor impulsiveness. These results are discordant with recent works [42] that report alcohol consumption is associated with total, motor, and cognitive impulsiveness.

However, the impulsivity results observed in the present study, considered as sensitivity to punishment and reward, are congruent with previous studies, since the sensitivity to reward, but not the sensitivity to punishment, seems to be related to alcohol consumption [21,31].

In short, according to our results and other studies, it can be concluded that the impulsive-disinhibited personality factor is strongly related to alcohol consumption. Interestingly, some dimensions of impulsivity are differentially related to different variables of alcohol consumption. Thus, for example, impulsivity seems to be more associated to alcohol-related problems, whereas sensation seeking is more related to a non-problematic use of alcohol [21].

Our results also indicate that not all dimensions of EI have the same relation to alcohol consumption. On the one hand, a negative correlation between emotional attention and the frequency of alcohol consumption, but not the amount of consumption, is observed. We did not found correlations between clarity and emotional repair regarding alcohol consumption.

These results differ partially from those described in previous works, in which emotional attention was shown to positively and directly correlate with alcohol consumption, and the emotional repair showed a negative correlation with alcohol consumption [30]. Thus, young people reporting not consuming alcohol consider that they pay less attention to their emotions and report a greater perceived ability to manage their emotional states, while young alcohol consumers show less emotional repair than non-consumers.

Data reported by Trinidad and Johnson in 2002 [25] seems to differ slightly different from our results. These authors observed significant correlations between emotional clarity and alcohol consumption. Other studies have not found significant relationships between emotional attention and the clarity and emotional reparation factors, although high clarity scores have correlated significantly with emotional reparation [24].

In general, the results of previous research show a negative and significant correlation between EI and alcohol consumption [27,28], indicating that a low EI is the best predictor of alcohol consumption. This relationship is based on the assumption that a high EI allows the person to have self-control and emotional management skills, which involve positive coping and better decision-making, which decreases the probability of consuming alcohol.

Taking into account impulsivity and EI, our results are congruent with others studies, showing that young people with low levels of EI tend to be more impulsive and have a worse handling of their emotions, a fact that can increase the risk of consumption, while young people with good emotional skills show lower substance use [25,30,46].

Our second objective was to determine if EI (attention, clarity and emotional repair) has a serial mediating effect on the relationship between the components of impulsivity and alcohol consumption. This potential effect was deduced from the hypothesis that greater impulsivity is associated with lower levels of emotional intelligence which, in turn, implies greater alcohol consumption, both in frequency and quantity. In this sense, the results of the mediation analyses show that disinhibition has a significant direct effect on the frequency of alcohol consumption, and also has an indirect effect, through emotional clarity, which in turn affects the frequency of consumption. These results partially differ from the initial hypothesis, since greater impulsivity is associated with lower levels of emotional clarity, which in turn implies lower alcohol consumption, but only regarding the frequency of consumption.

Therefore, EI seems to mediate the relationship between disinhibition and the frequency of alcohol consumption, thus confirming that EI may mediate the effects of impulsivity on substance use. These results are congruent with previous works showing that EI can explain and modulate the consumption of substances such as alcohol [47,48,49,50]. Thus, young people with lower levels of disinhibition, i.e., less impulsive, are expected to consume alcohol less frequently. But this relationship is affected by the understanding of young people of their own emotional states. In fact, if young people have emotional clarity and understand their own emotional states, more alcohol consumption becomes more likely, and it could be argued that in less disinhibited people alcohol consumption is a maladaptive mechanism to suffering or emotional states.

The uses of self-reporting measures is a limitation of this study. In general the levels of honesty may be compromised using self-reports. This applies particularly when assessing risky, sensitive or highly stigmatized behaviors such as drug use. Therefore, students might have insufficiently recorded the amount of alcohol consumption [51], although the validity in the alcohol use register may be considered high because we used closed questions [52].

## 5. Conclusions

To conclude, our results suggest that the impulsive-uninhibited personality factor is strongly related to alcohol consumption, but this relationship is mediated by EI. Overall, these results could be of great interest for the prevention of alcohol consumption in young people, considering that the skills attributed to EI are susceptible to improvement through learning. Therefore, we can assume that training young people in skills involved in emotional intelligence could provide strategies to protect themselves against harmful alcohol consumption.

## Figures and Tables

**Table 1:** Descriptive statistics and sex difference (Student’s *t*-test) for personality trait and emotional intelligence.

		Females		Males		Overall		*t*	*gl*	*p*
		M	*SD*	M	*SD*	M	*SD*			
Alcohol Frequency		46.39	42.25	57.85	54.84	48.33	44.76	−1.592	80.184	0.115
Amount of Alcohol		3.35	1.53	3.86	2.06	3.43	1.64	−1.911	78.966	0.060
SSS-V	Thrill and Adventure Seeking	5.52	2.96	6.94	2.74	5.76	2.97	−3.568	382	0.000 **
	Experience Seeking	6.34	1.76	6.55	1.99	6.37	1.80	−0.893	382	0.373
	Disinhibition	4.35	2.08	5.74	2.59	4.58	2.23	−4.073	81.603	0.000 **
	Boredom Susceptibility	3.57	1.96	3.60	1.93	3.57	1.96	−0.122	382	0.903
BIS-11	Cognitive	13.97	4.11	15.51	4.79	14.23	4.27	−2.672	382	0.008 **
	Motor	15.51	6.23	15.57	6.13	15.52	6.21	−0.065	382	0.948
	Non-planning	14.27	5.93	16.54	7.09	14.65	6.20	−2.714	382	0.007 **
SPSRQ	Sensitivity to Punishment	11.32	4.92	10.23	5.43	11.14	5.02	1.602	382	0.11
	Sensitivity to Reward	9.58	4.16	11.32	4.33	9.87	4.23	−3.065	382	0.002 **
TMMS-24	Emotional Attention	29.18	5.24	28.54	5.00	29.07	5.20	0.9	382	0.369
	Emotional Clarity	28.83	5.39	29.25	4.99	28.90	5.32	−0.569	382	0.57
	Emotional Repair	29.16	5.82	30.17	4.53	29.33	5.63	−1.560	111.846	0.122

Notes: ** *p* < 0.001. *N* = 384. Abbreviations: M = Means, *SD* = Standard Deviations. SSS-V, Sensation Seeking Scale; TMMS-24, Trait Meta-Mood Scale; BIS-11, Barratt Impulsivity Scales 11; SPSRQ, Sensitivity to Punishment and Sensitivity to Reward Questionnaire.

**Table 2:** Pearson correlations between alcohol, personality trait, emotional intelligence, and age.

		1	2	3	4	5	6	7	8	9	10	11	12	13	14
1	Alcohol Frecuency	-													
2	Amount of alcohol	0.260 **													
3	Thrill and Adventure Seeking	0.162 **	0.129 *												
4	Experience Seeking	0.180 **	0.172 **	0.376 **											
5	Disinhibition	0.312 **	0.262 **	0.356 **	0.492 **										
6	Boredom Susceptibility	0.070	0.095	0.195 **	0.181 **	0.290 **									
7	Cognitive	0.085	0.087	0.076	0.272 **	0.315 **	0.229 **								
8	Motor	0.117 *	0.114 *	0.119 *	0.259 **	0.313 **	0.314 **	0.493 **							
9	No planning	0.147 **	0.142 **	0.226 **	0.291 **	0.301 **	0.331 **	0.392 **	0.356 **						
10	Sensitivity to Punishment	−0.086	−0.082	−0.126 *	−0.151 **	−0.045	−0.034	0.051	−0.084	−0.112 *					
11	Sensitivity to Reward	0.186 **	0.131 **	0.189 **	0.237 **	0.508 **	0.353 **	0.289 **	0.388 **	0.183 **	0.008				
12	Emotional Attention	−0.101 *	−0.042	−0.037	−0.001	0.009	−0.059	0.035	0.014	−0.028	0.238 **	0.064			
13	Emotional Clarity	0.029	0.010	0.023	−0.105 *	−0.182 **	−0.219 **	−0.208 **	−0.182 **	−0.083	−0.319 **	−0.183 **	0.116 *		
14	Emotional Repair	−0.012	−0.043	0.250 **	0.053	0.030	−0.102 *	−0.056	0.011	0.012	−0.339 **	−0.025	0.017	0.274 **	
15	Age	−0.059	0.056	0.003	0.097	0.065	−0.056	−0.006	−0.034	−0.050	−0.050	−0.038	0.005	0.125 *	−0.023

Notes: * *p* < 0.05; ** *p* < 0.001. *N* = 384.

**Table 3:** Linear multiple regression analyses predicting alcohol variables.

	**Alcohol Frequency**											
	**Model 1**			**Model 2**			**Model 3**			**Model 4**		
*R*	0.363			0.363			0.343			0.344		
*R* ^2^	0.132			0.132			0.118			0.118		
*F*-value	4.692 **			4.320 **			16.939 **			12.685 **		
Predictor variables	Emotional Attention	Emotional Clarity	DIS	Emotional Attention	Emotional Clarity	DIS	Emotional Attention	Emotional Clarity	DIS	Emotional Attention	Emotional Clarity	DIS
*Beta*	−0.114	0.11	0.273	−0.114	0.11	0.273	−0.116	0.102	0.331	−0.116	0.102	0.329
*t*	−2.209 *	1.959 *	4.199 **	−2.202 *	1.953 *	4.123 **	−2.398 *	2.077 *	6.758 **	−2.378 *	2.049 *	6.489 **
	**Amount of Alcohol**											
	**Model 1**	**Model 2**	**Model 3**	**Model 4**								
*R*	0.301	0.306	0.262	0.268								
*R* ^2^	0.091	0.093	0.069	0.072								
*F*-value	3.077 **	2.931 **	28.173 **	14.797 **								
Predictor variables	DIS	DIS	DIS	DIS								
*Beta*	0.229	0.217	0.267	0.248								
*t*	3.446 **	3.213 **	5.308 **	4.887 **								

Notes: ** *p* < 0.001; * *p* < 0.05. *N* = 384. Predictors: model 1 = all subscales of impulsivity and emotional intelligence; model 2 = all subscales and sex; model 3 = significant subscales of model 2; model 4 = model 3 and sex.

**Table 4:** Path coefficients; total effect; direct effect; indirect effect; specific indirect effects and 95% bias-corrected confidence interval predicting frequency alcohol scores (*N* = 384).

Path	Coefficient	SE	BootLLCI	BootULCI	*t*	*p*
Total effect (*c*)	6.255	0.9762	4.336	8.175	6.408	0.000
Direct effect (*c*′)	6.738	0.987	4.796	8.680	6.822	0.000
*a_1_*	0.0215	0.1193	−0.2131	0.256	0.1798	0.857
*a_2_*	−0.4368	0.1194	−0.6716	−0.202	−36.576	0.000
*a_3_*	0.2089	0.1263	−0.0394	0.4573	1.654	0.098
*b_1_*	−1.009	0.4179	−1.831	−0.1882	−2.416	0.016
*b_2_*	0.9897	0.4328	0.1387	1.841	2.287	0.022
*b_3_*	−0.4153	0.3997	−1.201	0.3706	−1.039	0.299
*d*_21_	0.1204	0.0512	0.0197	0.2211	2.351	0.019
*d*_31_	−0.0188	0.0536	−0.1242	0.0867	−0.35	0.726
*d*_32_	0.3078	0.0533	0.2031	0.4126	5.779	0.000
Indirect effects	**Effect**	**SE**	**BootLLCI**	**BootULCI**		
Total indirect effect	−0.4825	0.2643	−1.048	−0.0021		
*Ind2: a_2_b_2_*	−0.4323	0.2293	−0.9312	−0.0417		

Notes: Abbreviations: BootLLCI, bootstrapping lower limit confidence interval; BootULCI, bootstrapping upper limit confidence interval; SE, standard error. Model: 6. Y: Alcohol Frequency. X: Disinhibition. M1: Emotional attention. M2: Emotional clarity.M3: Emotional repair. *N* = 384.
